# Automatic protective ventilation using the ARDSNet protocol with the additional monitoring of electrical impedance tomography

**DOI:** 10.1186/cc13937

**Published:** 2014-06-23

**Authors:** Anake Pomprapa, David Schwaiberger, Philipp Pickerodt, Onno Tjarks, Burkhard Lachmann, Steffen Leonhardt

**Affiliations:** 1 Philips Chair of Medical Information Technology, Helmholtz-Institute for Biomedical Engineering, RWTH Aachen University, Pauwelsstrasse 20, Aachen 52074, Germany; 2 Department of Anesthesiology and Intensive Care Medicine, Campus Charité Mitte and Campus Virchow-Klinikum, Charité – University Medicine Berlin, Campus Virchow-Klinikum, Augustenburger Platz 1, Berlin 13353, Germany

## Abstract

**Introduction:**

Automatic ventilation for patients with respiratory failure aims at reducing mortality and can minimize the workload of clinical staff, offer standardized continuous care, and ultimately save the overall cost of therapy. We therefore developed a prototype for closed-loop ventilation using acute respiratory distress syndrome network (ARDSNet) protocol, called autoARDSNet.

**Methods:**

A protocol-driven ventilation using goal-oriented structural programming was implemented and used for 4 hours in seven pigs with lavage-induced acute respiratory distress syndrome (ARDS). Oxygenation, plateau pressure and pH goals were controlled during the automatic ventilation therapy using autoARDSNet. Monitoring included standard respiratory, arterial blood gas analysis and electrical impedance tomography (EIT) images. After 2-hour automatic ventilation, a disconnection of the animal from the ventilator was carried out for 10 seconds, simulating a frequent clinical scenario for routine clinical care or intra-hospital transport.

**Results:**

This pilot study of seven pigs showed stable and robust response for oxygenation, plateau pressure and pH value using the automated system. A 10-second disconnection at the patient-ventilator interface caused impaired oxygenation and severe acidosis. However, the automated protocol-driven ventilation was able to solve these problems. Additionally, regional ventilation was monitored by EIT for the evaluation of ventilation in real-time at bedside with one prominent case of pneumothorax.

**Conclusions:**

We implemented an automatic ventilation therapy using ARDSNet protocol with seven pigs. All positive outcomes were obtained by the closed-loop ventilation therapy, which can offer a continuous standard protocol-driven algorithm to ARDS subjects.

## Introduction

Acute respiratory distress syndrome (ARDS) is a severe form of acute multifactorial lung injury with acute hypoxic respiratory failure. Despite extensive information on ARDS regarding its clinical features [1-4], pathologic findings [5] and prognosis [6,7], the mortality has remained unchanged for many decades [8,9]. A considerable challenge in critical care medicine is therefore to rescue ARDS patients by means of ventilatory therapy in the short term and to optimize morbidity in the long term [10].

The term ARDS was first introduced in 1967 [11]. However, a clear definition was not quantified and remained controversial for many decades [12]. In 1994, a standard definition was recommended by the American–European Consensus Conference committee [13]. The pathological state of ARDS was defined by the ratio of arterial oxygen tension (PaO_2_) and fraction of inspired oxygen (FiO_2_) (PaO_2_/FiO_2_ < 200) in the presence of bilateral infiltrates on the chest X-ray image and pulmonary artery wedge pressure ≤ 18 mmHg. In 2012, the definition of ARDS was revised and called the Berlin definition [14,15]. The criteria were similar to those of the American–European Consensus Conference, but with further classification of the severity; that is, mild (200 < PaO_2_/FiO_2_ ≤ 300), moderate (100 < PaO_2_/FiO_2_ ≤ 200) and severe (PaO_2_/FiO_2_ ≤ 100) at positive end-expiratory pressure (PEEP) ≥ 5 cmH_2_O [15].

ARDS is caused by the formation of protein-rich alveolar edema after damage to the integrity of the alveolar–capillary barrier [16]. Patients with ARDS generally experience shortness of breath with loss of lung compliance due to the formation of noncardiogenic pulmonary edema and inactivation of surfactant, leading to alveolar collapse and atelectasis. Progressive hypoxia and an increased work of breathing are then unavoidable. At the onset, ventilation support with mechanical ventilation is generally required.

Since 2000, protective ventilation using the Acute Respiratory Distress Syndrome Network (ARDSNet) protocol has continued to be the cornerstone in intensive care for ARDS therapy. With this ventilation strategy, a reduction of mortality (31% vs. 39.8%) was clearly demonstrated as compared with conventional ventilation using a higher tidal volume per kilogram of predicted body weight (PBW) of 12 ml/kg [17]. With this outstanding result, the original ARDSNet protocol was implemented in our automatic ventilation therapy system. The therapeutic approach focuses not only on avoiding ventilator-induced lung injury by using low tidal volume per PBW (≤6 ml/kg) and restricted plateau pressure (P_plat_ ≤30 cmH_2_O), but also on providing sufficient gas exchange with defined oxygenation targets and meeting pH goals. The goals for protective ventilation using this protocol are to improve and regulate oxygenation, to minimize P_plat_ and to control pH value. Five ventilatory settings are of particular interest: FiO_2_, PEEP, tidal volume (V_T_), respiratory rate (RR) and inspiratory–expiratory time ratio (I:E ratio).

To achieve these goals, the ventilation variables have to be properly adjusted during ventilatory therapy, which generally requires continuous care from clinical staff, especially for patients with severe ARDS. To minimize the workload of daily clinical practice and to maintain the standard protocol of protective ventilation, we introduce automatic control of ventilation as a concept for the treatment. In this article, we describe a strategy of automatic ventilation in ARDS patients, including continuous monitoring of regional ventilation at thoracic cavity using electrical impedance tomography (EIT) images [18]. All therapeutic decisions in this context rely on the original ARDSNet protocol.

## Materials and methods

In this section, we describe the concept for automatic ventilation using the ARDSNet protocol and present some details on the implementation.

### System setup

The system consists of a panel PC (PPC-154 T; Advantech Co., Ltd, Taipei, Taiwan), a mechanical ventilator (SERVO 300; Marquet Critical Care AB, Solna, Sweden), and other equipment including a capnography device with pulse oximetry (CO_2_SMO+; Philips Respironics, Best, The Netherlands), a spectrophotometry device (CeVOX; Pulsion Medical Systems SE, Feldkirchen, Germany) to measure arterial oxygen saturation (SaO_2_), a patient monitor (Sirecust 960; Siemens AG, Munich, Germany), and an EIT device (GOE-MF II; Dräger AG, Lübeck, Germany). All measured signals are transmitted directly to the panel PC including parameters from the mechanical ventilator, by which airway pressure and airway flow are converted by a 12-bit analog-to-digital converter (KPCMCIA-12AI-C; Keithley Instruments Inc., Cleveland, OH, USA). The computed commands of ventilatory variables are transmitted from the panel PC to the mechanical ventilator by a 12-bit digital-to-analog converter (PCMDA12B; SuperLogics Inc., Waltham, MA, USA). Automatic adjustment of ventilatory settings can be made by this setup.

### Communication protocol

The protocols from the different commercial devices are graphically programmed using Labview software (version 7.1; National Instruments Inc., USA). The specific binary codes for each device are transmitted from the panel PC to the devices. The interfaces are based on RS-232 standard. To obtain the up-to-date measured parameters, the requested commands must be repeatedly sent to all devices in every sampling period of 100 milliseconds. Once the panel PC receives a response from a device, the data are decoded and saved on a regular basis within the sampling time.

### Preparation for animal studies

After approval from the Department of Health and Social Services Berlin (reference number IC 113-G0151/10), all animal procedures were conducted complying with national regulations and institutional animal care committee guidelines. Seven female domestic pigs (29 ± 3 kg) received premedication and general anesthesia with thiopental, fentanyl and pancuronium, and were then tracheotomized in a supine position. A spectrophotometry catheter (CeVOX; Pulsion Medical Systems AG) was inserted into the carotid artery for measuring SaO_2_. Noninvasive measurements of peripheral oxygen saturation (SpO_2_) from capnography device and from the patient monitor were placed at the left ear and at the tail, respectively. In addition, a central venous line and a pulmonary artery catheter were placed into the internal jugular vein for continuous monitoring of pulmonary artery pressure and central venous pressure and for drug and crystalloid fluid infusion. Subsequently, the pigs underwent surfactant depletion with repetitive lavages by warm (37 to 38°C) saline solution (0.9% NaCl, 40 ml/kg body weight) to induce ARDS (PaO_2_/FiO_2_ < 200 mmHg) at FiO_2_ of 1.0 [19]. The lavages were carried out between two and four times (average three times) within 5 minutes. The pigs were then ventilated in volume-controlled mode with V_T_ of 6 ml/kg body weight and static PEEP of 5 cmH_2_O. After 30 minutes, the closed-loop ventilation was started. However, after 2 hours of ventilation, a disconnection from the ventilator was performed for 10 seconds, simulating involuntary patient–ventilator disconnection during patient handling.

### ARDSNet protocol

To some extent, our algorithm of the ARDSNet protocol is based on earlier work of our group [20,21]. The protocol is a ventilation strategy using low tidal volume at 6 ml/kg of the PBW based on the formulae given in Equations (1) and (2) for male and female subjects [17]:

(1) $$\text{PBW}\;\left(\text{male}\right)=50+2.3\left(\text{height}\;\left[\text{inches}\right]-60\right)$$

(2) $$\text{PBW}\;\left(\text{female}\right)=45.5+2.3\left(\text{height}\;\left[\text{inches}\right]-60\right)$$

In the present study, PBW was replaced with the actual measured body weight for the ventilation setting on V_T_. The following goals should be fulfilled.

#### Oxygenation goal: PaO_2_ for 55 to 80 mmHg or SpO_2_ for 88 to 95%

A linear combination between PEEP and FiO_2_, shown in Table 1 for lower PEEP/higher FiO_2_, was applied for control of oxygenation.

**Table 1:** **PEEP and FiO**_**2 **_**combination from the ARDSNet protocol (lower PEEP/higher FiO**_**2**_**)**

PEEP (cmH_2_O)	5	5	8	8	10	10	10	12	14	14	14	16	18	18 to 24
FiO_2_	0.3	0.4	0.4	0.5	0.5	0.6	0.7	0.7	0.7	0.8	0.9	0.9	0.9	1.0

ARDSNet, Acute Respiratory Distress Syndrome Network; FiO_2_, fraction of inspired oxygen; PEEP, positive end-expiratory pressure.

Generally, PEEP is used to prevent lung collapse, while more FiO_2_ is given in order to meet the predefined oxygenation goal. The increment of both ventilation variables improves oxygenation for ARDS patients. Hence, in our implementation, the lower PEEP/higher FiO_2_ table of the protocol [17] was used for the automatic ventilation.

#### ***Plateau pressure goal: ≤ 30 cmH***_***2***_ ***O***

Based on the recommendation, P_plat_ should be checked at least every 4 hours and also after the change of PEEP or V_T_. In our automatic ventilation scheme, P_plat_ was automatically checked every 10 minutes. The inspiratory pause was set to 0.5 seconds for five consecutive breaths, and the average of P_plat_ from these breaths was used to represent the measured P_plat_. Further corrective action for V_T_ adjustment is carried out by the following rules: if P_plat_ > 30 cmH_2_O, tidal volume per weight (V_TPW_) may be decreased by 1 ml/kg with the minimum value of 4 ml/kg; and if P_plat_ < 25 cmH_2_O and V_TPW_ < 6 ml/kg, V_TPW_ may be increased by 1 ml/kg until P_plat_ > 25 cmH_2_O or V_TPW_ = 6 ml/kg.

Hence, V_TPW_ was generally set to 6 ml/kg but was allowed to be reduced to 4 or 5 ml/kg if P_plat_ > 30 cmH_2_O. On the other hand, V_TPW_ was allowed to be 7 or 8 ml/kg if breath stacking or dyssynchrony was observed during the ventilation therapy. For the implementation, P_plat_ was regularly evaluated about every 10 minutes.

#### ***pH goal: 7.30 to 7.45***

Arterial pH values were measured by arterial blood gas (ABG) analysis (ABL 5; Radiometer Copenhagen, Copenhagen, Denmark) every 30 minutes; this value was manually entered into the panel PC for further evaluation of the pH goal. The initial RR was set to approximate baseline minute ventilation, but should be limited by a maximum value of 35 breaths per minute (bpm). The initial setting of RR was computed as follows:

(3) $$\mathrm{RR}=\frac{MV_{\text{baseline}}}{V_{\text{TPW}}\times \text{Weight}}$$

Based on the averaged body weight of the pigs (29 ± 3 kg), baseline minute ventilation of 4 l/minute was chosen for the initial setting of RR. Further adjustment of RR was based on the measured pH value by the resulting acidosis or alkalosis.

#### ***Rules for acidosis management (pH < 7.30)***

The following rules were followed for management of acidosis: if pH = 7.15 to 7.30, RR should be increased until pH > 7.30 or PaCO_2_ < 25 mmHg (maximum RR = 35 bpm); if pH < 7.15, RR should be increased to 35 bpm; and if pH remains < 7.15, V_TPW_ should be increased by 1 ml/kg until pH > 7.15 (maximum V_TPW_ = 8 ml/kg).

#### ***Rules for alkalosis management (pH > 7.45)***

If pH > 7.45, RR should be decreased. A stepwise change of RR is set at ±5 bpm with the aim to control pH value.

#### ***Inspiratory–expiratory ratio goal***

Generally, the I:E ratio for ARDS patients is set between 1:1 and 1:3. In the present study, the I:E ratio was fixed at 1:2.

### Programming architecture

**Figure 1:**
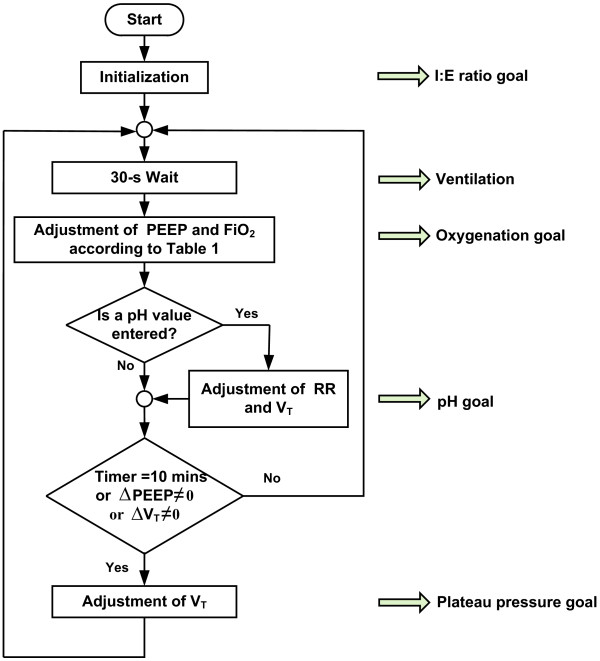
**Flowchart for automatic ventilation using the ARDSNet protocol.** ARDSNet, Acute Respiratory Distress Syndrome Network; FiO_2_, fraction of inspired oxygen; I:E ratio, inspiratory–expiratory ratio; PEEP, positive end-expiratory pressure; RR, respiratory rate; V_T_, tidal volume.

The protocol can be effectively developed by goal-oriented structural programming. The overall complexity of the protocol is simplified by a task-based programming structure, presented in Figure 1. This structure increases efficiency in coding the program.

Once the automatic ventilation is started, the initialization activates all initial settings of ventilation variables, such as PEEP, FiO_2_, V_T_, RR, and I:E ratio. Regarding the oxygenation goal, PEEP of 14 cmH_2_O and FiO_2_ of 0.7 from the middle of the Table 1 are initially selected. These settings should be given for a number of breaths and this is represented by a 30-second waiting block or the ventilation task in Figure 1. Thereafter, SaO_2_ is evaluated for further adjustment of the PEEP and FiO_2_ combination.

During the experiments, a number of choices for oxygenation goal can be made either by invasive measurement of SaO_2_ from the spectrophotometry device (CeVOX; Pulsion Medical Systems SE), or by noninvasive measurement of SpO_2_ from the capnography device (CO_2_SMO+; Philips Respironics), or from the patient monitor (Sirecust 960; Siemens AG). In our experiments, SaO_2_ measured from CeVOX was chosen for control of oxygenation. If SaO_2_ falls below 88%, a higher combination of PEEP and FiO_2_ should be given. In contrast, if SaO_2_ rises above 95%, a lower combination of PEEP and FiO_2_ should be applied to minimize hemodynamic effects of PEEP and to reduce the risk of oxygen toxicity.

After achieving the oxygenation goal, the next step is to check for the pH goal. If there is no new pH value, a further evaluation of P_plat_ should be made. However, if a new pH value is manually given, proper adjustment of RR and V_T_ should be performed for the pH goal. After fulfilling the pH goal, the plateau pressure goal is carried out every 10 minutes or after a change in PEEP or a change in V_T_. All new variables are applied to the subject for a number of breaths. This repeated process is continuously performed. Using this goal-oriented structure, all goals of the ARDSNet protocol will be accomplished.

## Results

Employing the porcine model of induced surfactant depletion as previously described, we now present the results of the automatic ventilation therapy using the Acute Respiratory Distress Syndrome Network (autoARDSNet) protocol, based on invasive measurement of SaO_2_ in the carotid artery, with additional EIT images.

### Oxygenation goal during the autoARDSNet protocol

Seven cases of porcine dynamics were studied with the protocol. As an example of the system performance, we present 4 hours of ventilation from one of the pigs (27 kg). During the lavage, the ventilation settings were set by manual operation and the automatic mode was subsequently turned on for ventilation therapy after PaO_2_/FiO_2_ < 200 mmHg for 15 minutes. The oxygenation was kept within the range between 88 and 95%, hence satisfying the oxygenation criterion. Using Table 1, the knowledge-based controller was able to stabilize and regulate the SaO_2_ value.

**Figure 2:**
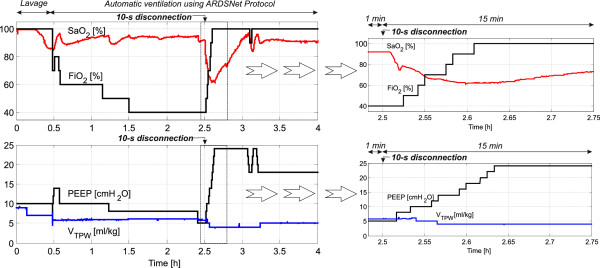
**Control of arterial oxygen saturation using Table**1**for a 27 kg pig.** Right: magnified view for 15 minutes after the disconnection time (2.5 hours). ARDSNet, Acute Respiratory Distress Syndrome Network; FiO_2_, fraction of inspired oxygen; SaO_2_, arterial oxygen saturation; PEEP, positive end-expiratory pressure; V_TPW_, tidal volume per weight.

Figure 2 shows the response of lung lavage in the first 30 minutes and the automatic ventilation for stabilization and regulation of SaO_2_ by adjusting PEEP and FiO_2_ referred to in Table 1. At 2.5 hours, or 2 hours after automatic ventilation, a disconnection of ventilation was made for 10 seconds, simulating a clinical scenario of airway suction or accidental disconnection. The controller was able to recover the critical situation of low oxygenation by step-by-step change for the values of PEEP and FiO_2_, until PEEP of 24 cmH_2_O and FiO_2_ of 1.0. Subsequently, an automatic titration of suitable PEEP and FiO_2_ was carried out again to fulfill the oxygenation goal.

### Plateau pressure during the autoARDSNet protocol

**Figure 3:**
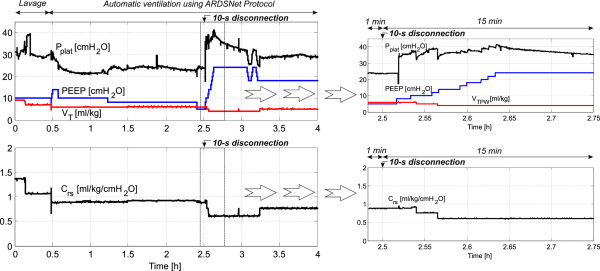
**Control of plateau pressure with the computed lung compliance for the 27 kg pig.** ARDSNet, Acute Respiratory Distress Syndrome Network; C_rs_, lung compliance; PEEP, positive end-expiratory pressure; P_plat_, plateau pressure; V_TPW_, tidal volume per weight.

To minimize ventilator-induced lung injury, the P_plat_ goal should be kept below 30 cmH_2_O by the adjustment of V_T_[13]. During the time 0.5 to 2.5 hours shown in Figure 3, P_plat_ was definitely less than 25 cmH_2_O while V_TPW_ was maintained at 6 ml/kg.

During the time between 2.5 and 3.25 hours (hypoxia as presented in Figure 2 due to disconnection at the patient–ventilator interface at 2.5 hours), P_plat_ was >30 cmH_2_O and V_TPW_ was reset from 6 to 5 ml/kg and from 5 to 4 ml/kg, respectively, while the PEEP and FiO_2_ combination was increased to possible maximum values. At 3.25 hours, when P_plat_ < 25 cmH_2_O and V_TPW_ < 6 ml/kg, V_TPW_ was automatically increased stepwise by 1 ml/kg increments to 5 ml/kg. Using this approach, the goal of P_plat_ is satisfied with the main objective to minimize P_plat_ ≤ 30 cmH_2_O.

### pH goal during the autoARDSNet protocol

**Figure 4:**
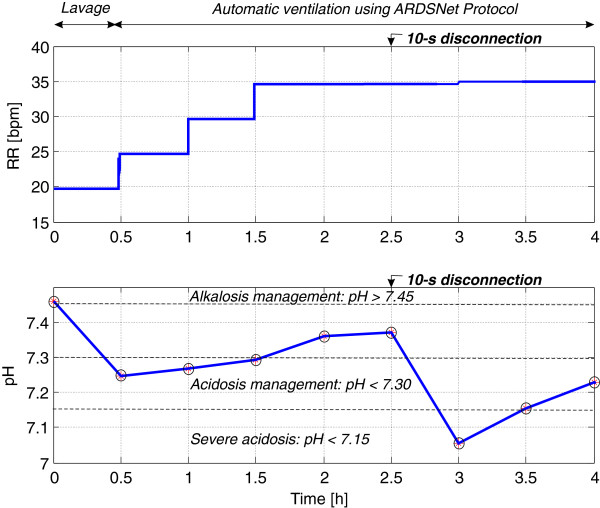
**Control of the pH value for the 27 kg pig.** Circles on the pH curve indicate manual arterial blood gas measurements. ARDSNet, Acute Respiratory Distress Syndrome Network; RR, respiratory rate.

Figure 4 shows the result of pH control for the 27 kg pig. The pH values were regularly measured every 30 minutes. At 0.5, 1 and 1.5 hours, the pH value was <7.30, and RR was increased by 5 bpm after entering the pH value into the system. At 2 and 2.5 hours, the pH goal was satisfied and RR remained unchanged. At 2.5 hours, ABG was measured before the 10-second disconnection at the patient–ventilator interface. At the next ABG (3 hours), the pH value falls below 7.15. RR was immediately set at 35 bpm to treat severe acidosis due to disconnection at the patient–ventilator interface. With the maximum limit of RR at 35 bpm, this resulted in an increase of the pH value to >7.15. At 3.5 and 4 hours, the pH value was 7.15 to 7.30 and RR should be increased. However, RR was already at its limit set at 35 bpm. 

### Monitoring of carbon dioxide

**Figure 5:**
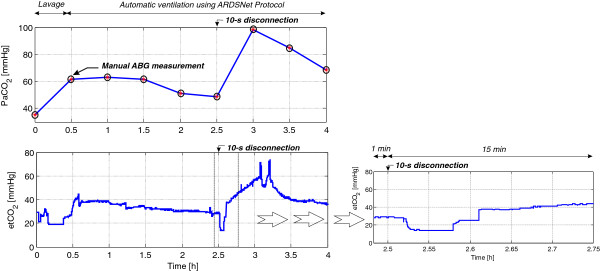
**Monitoring of arterial carbon dioxide tension and end-tidal carbon dioxide during automatic ventilation therapy using ARDSNet protocol for the 27 kg pig.** ABG, arterial blood gas; ARDSNet, Acute Respiratory Distress Syndrome Network; etCO_2_, end-tidal carbon dioxide; PaCO_2_, arterial carbon dioxide tension.

Figure 5 shows arterial carbon dioxide tension (PaCO_2_) from ABG and end-tidal carbon dioxide (etCO_2_) during the 4 hours of ventilation. PaCO_2_ significantly increased after lung lavage, which indicates poor gas exchange or partial lung collapse (atelectasis). This is also confirmed by the SaO_2_ curve and the EIT images in the next subsection. After turning on the protocol for 2 hours during 0.5 to 2.5 hours, gas exchange was gradually improved due to the ventilation therapy. Again, at 2.5 hours, poor gas exchange recurred during hypoxia because of disconnection at the patient–ventilator interface for 10 seconds. Since PaCO_2_ was taken before disconnection at the patient–ventilator interface, severe hypercapnia was later detected (at 3 hours). However, the automatic ventilation improved gas exchange and severe hypercapnia was relieved in a timely manner.

By monitoring carbon dioxide parameters, physiological dead space can be estimated by Bohr–Enghoff’s equation [22]:

(4) $$\frac{V_{D}}{V_{T}}=\frac{\text{PaC}O_{2}-\text{etC}O_{2}}{\text{PaC}O_{2}}$$

where V_D_ denotes physiological dead space. Based on the dataset of 4-hour ventilation, the average fraction of physiological dead space for this pig was 0.39. In the other words, approximately 60% of the tidal volume took part in the gas exchange.

### Electrical impedance tomography

**Figure 6:**
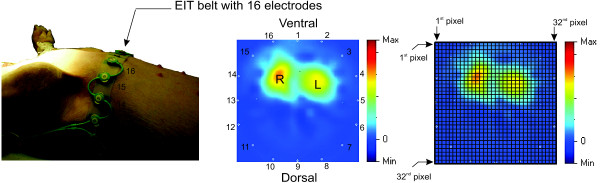
**Orientation of electrical impedance tomography belt with 16 electrodes and the reconstructed 32 pixel × 32 pixel EIT image after induction of ARDS in the rainbow-color coding scheme.** ARDS, Acute Respiratory Distress Syndrome; EIT, electrical impedance tomography; L, left; R, right.

**Figure 7:**
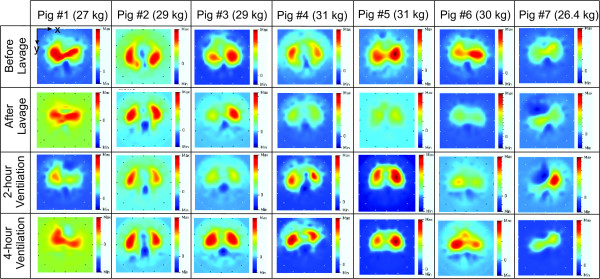
**Electrical impedance tomography images at the end of inspiration from seven pigs during automatic ventilation therapy using the ARDSNet protocol.** EIT, electrical impedance tomography.

EIT allows non-invasive monitoring of electrical impedance within the thoracic cavity in a two-dimensional and cross-sectional plane in order to assess regional ventilation [23]. Pathophysiological changes of the lung can be observed from the EIT images in real time at the bedside. Sixteen electrodes were used for the voltage measurement and the backprojection algorithm [24] was implemented for image reconstruction. A 32 pixel × 32 pixel EIT image is captured at the end of inspiration, as shown in Figure 6. Based on the attachment of the EIT belt in the predefined arrangement of the electrodes shown in Figure 6 (left image), ventral and dorsal parts of the animal are situated at the top (electrode position 1) and at the bottom (electrode position 9) of the EIT image, respectively. The position of the left and right lungs can therefore be determine in the specified position as shown and similar to the standard interpretation, obtained from a computed tomography scan image.All seven female pigs (weighing 29 ± 3 kg) were ventilated using the autoARDSNet protocol; the results of their EIT images are summarized in Figure 7. These results show the EIT images before and after lavage, and after 2 hours and 4 hours of ventilation using the protocol. The area of high electrical impedance corresponds to the movement of air, which is designated by tones of orange and yellow. After lavage, a loss of lung volume and poor dorsal ventilation can be observed by the images in all cases. After 2 hours and 4 hours of ventilation, a progressive improvement of dorsal ventilation can be seen compared with the EIT images after lavage.

Regarding pig #7, pneumothorax was observed by EIT image after 2 hours of ventilation demonstrating that only the left lung was ventilated. A corrective action was made at 2.75 hours to release excess pressure at the right lung, which improved lung compliance, oxygenation, hemodynamics and carbon dioxide exchange. Based on this experience, we believe that the EIT device is useful for practical decision-making at the bedside.

**Figure 8:**
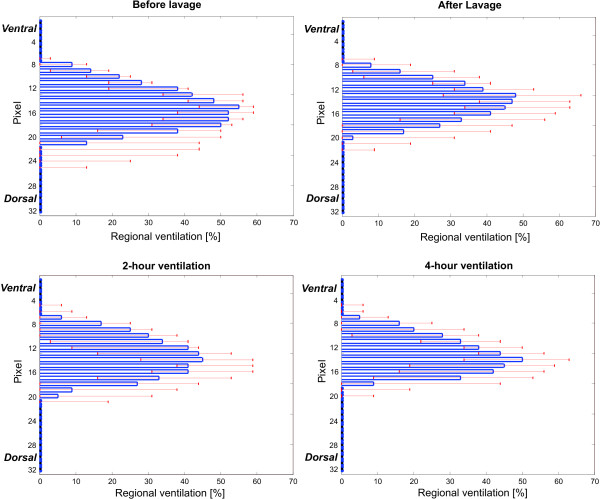
Regional analysis of ventilation for six pigs (excluding pig #7).

Regional analysis of ventilation [25] was carried out for six pigs (excluding pig #7 due to the pneumothorax), as shown in Figure 8. Horizontal bars represent the median of regional ventilation in percent at each specific pixel, while the whiskers are the outliers of extreme regional ventilation. Before lavage, median regional ventilation at the 15th pixel contributed the most to ventilation (55%). After lavage, the 13th pixel occupied the leading median regional ventilation of 48%, reflecting atelectasis in dorsal lung sections. After 2 hours and 4 hours of automated ventilation, the 14th pixel contributed the most to median regional ventilation of 45% and 50%, respectively, signifying the recruitment of previously atelectatic surface.

### Significant parameters during the autoARDSNet protocol

**Figure 9:**
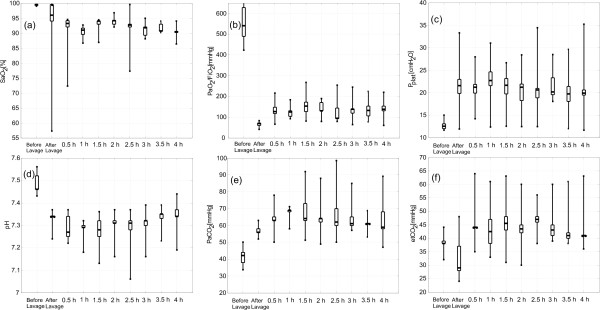
**Box-and-whisker plots for significant parameters during automatic ventilation therapy using the Acute Respiratory Distress Syndrome Network protocol. (a)** Arterial oxygen saturation (SaO_2_). **(b)** Arterial oxygen tension/fraction of inspired oxygen (PaO_2_/FiO_2_). **(c)** Plateau pressure (P_plat_). **(d)** pH. **(e)** Arterial carbon dioxide tension (PaCO_2_). **(f)** End-tidal carbon dioxide (etCO_2_).

Box-and-whisker plots indicating the median (25th to 75th percentiles) are shown in Figure 9. These plots quantitatively describe various significant parameters for all seven pigs. The parameters are presented before lavage, after lavage and every 0.5 hours. During the process of lavage inducing ARDS, PaO_2_/FiO_2_ was evaluated by ABG. The median of PaO_2_/FiO_2_ was 70 mmHg and all cases were below 100 mmHg, representing severe ARDS. By regulating SaO_2_ at 88 to 95% in Figure 9a, PaO_2_/FiO_2_ values were improved for all cases by the protocol as shown in Figure 9b. At 2.5 hours, disconnection at the patient–ventilator interface was carried out and the median of PaO_2_/FiO_2_ was 94 mmHg. The 4 hours of ventilation using the protocol increased PaO_2_/FiO_2_ and the ARDS condition generally improved from severe ARDS to moderate ARDS based on the Berlin definition [15].

In Figure 9c, the P_plat_ goal ≤30 cmH_2_O was satisfied in most cases. However, the pH value in Figure 9d was slightly lower than the pH goal between 7.30 and 7.45 in the first 1.5 hours of ventilation period for the protocol. Initial minute ventilation or baseline minute ventilation may increase from 4 to 5 l/minute to improve the pH value at the beginning of automatic ventilation.

Based on PaCO_2_ measured from ABG every 0.5 hours, Figure 9e represents permissive hypercapnia with an approximate value of 60 mmHg. Whilst Figure 9f shows etCO_2_ of an average 43 mmHg during ventilation therapy, etCO_2_ differed significantly from PaCO_2_, indicating a diffusion problem.

## Discussion

To achieve the oxygenation goal, two parameters (PaO_2_ in the range 55 to 80 mmHg and SaO_2_ between 88 and 95%) can be selected for the control objective. PaO_2_ was not chosen for this control objective because no commercial device for continuous measurement currently exists. In our setup, a change in PEEP and FiO_2_ translates to changes of SaO_2_ at the carotid artery in about 7 seconds, whereas it takes about 40 seconds for a change of SpO_2_ at the pig tail. SaO_2_ would also be valid even if circulation centralizes. However, technically both the SaO_2_ and the SpO_2_ signals can be used in our system. Because of the more stable and faster SaO_2_ response time, we chose this signal for establishing our automatized ARDSNet protocol. From a clinical perspective, SaO_2_ measurements will need to be replaced by high-quality SpO_2_ measurements.

PEEP also plays a vital role to prevent atelectasis. Several tables for lower and higher PEEP have been proposed as a guideline for ventilating a patient [17,18]. However, the proposed PEEP value might not be optimal: if PEEP is too high, it will cause regional hyperinflation: if PEEP is too low, it will cause dorsal end-expiratory collapse and excessive cyclic shear forces between atelectatic and nonatelectatic areas [26]. To optimize PEEP, many titration techniques have been developed; for instance, using the stress index [27,28], the assessment of transpulmonary pressure [29], or the optimization of ventilation homogeneity by EIT [30]. Owing to possible oxygen toxicity by excess FiO_2_[31], a feedback control system for regulating oxygenation to prespecified targets using FiO_2_[32] may thus use any combination of the protocols described above for PEEP titration. Hyperoxia shall then be avoided. Hence, many options to reach the oxygenation goal are available for a new formulation of PEEP–FiO_2_ combination.

P_plat_ was measured during an end-inspiratory pause for 0.5 seconds and represents alveolar pressure. P_plat_ > 30 cmH_2_O is associated with a higher mortality rate [17]. To fulfill the second goal of protective ventilation, V_TPW_ should be reduced in stepwise increments of 1 cmH_2_O, with a lowest limit of 4 ml/kg. For severe ARDS patients, PEEP should be set relatively high enough to satisfy oxygenation goal. Hence, V_TPW_ should automatically be minimized to either 5 or 4 ml/kg, so that P_plat_ is forced to be less than 30 cmH_2_O. During ventilation therapy using the autoARDSNet protocol, P_plat_ may not be able to meet the goal at all times. With automatic ventilation, P_plat_ was regularly evaluated and discrete (sampling) control of the P_plat_ value is implemented.

The pH goal can be achieved by adjusting of RR and V_T_ based on ABG taken every 30 minutes. With this time frame, perfect control of the pH value may not always be satisfied. Better control of the pH value can (theoretically) be improved by introducing continuous measurement of the pH value; however, no device is currently available to achieve this. Additionally, during the animal studies, an I:E ratio of 1:2 was fixed for the entire period of ventilation. A change of I:E ratio can influence carbon dioxide elimination [33]. For an adult, a range of 1:1 to 1:3 is considered acceptable for mechanical ventilation. Further studies are needed to examine changes in the I:E ratio and their correlation with the pH goal during automatic ventilation therapy. Carbon dioxide control based on adjustment of the I:E ratio would add another dimension of algorithmic flexibility; however, it would also increase the complexity of the rule base.

The three main goals, namely oxygenation, pH and plateau pressure, are targeted during the therapy. The oxygenation goal is considered highest priority, and in our algorithm the evaluation of this goal is carried out every 30 seconds. Owing to a rather slow response of the underlying physiological dynamics, the delay time of 30 seconds was chosen for acquiring the SaO_2_ response for a particular setting of ventilatory variables. Plateau pressure and pH goals were considered of secondary and tertiary priority and thus their evaluation periods were chosen to be 10 minutes and 30 minutes, respectively. Remember that in the original ARDSNet protocol the advice was to measure P_plat_ at least every 4 hours [17]. For the pH goal, measurements were based on 30-minute ABG analysis.

The monitoring of carbon dioxide (PaCO_2_ and etCO_2_) provides useful physiological information for gas exchange and physiological dead space. During 4 hours of ventilation, dead space was relatively constant, and in such cases etCO_2_ could be used to estimate PaCO_2_, even in the case of inhomogeneous lung condition in such cases as ARDS. The adjustment of RR therefore leads to a regulation of etCO_2_ and PaCO_2_[34]. Based on a mass balance, Equation (4) assumes that all the expired carbon dioxide comes from the alveolar gas. For the impaired alveolar gas exchange, there are a number of possible causes; for example, perfusion deficiency, diffusion barriers due to pulmonary edema, and reduced alveolar ventilation due to bronchial obstruction [35]. All of these problematic sources influence the computation of dead space. Using this animal model, dead space calculation reflects true dead space before the lavage, but after the lavage this computation may reveal only impaired diffusion in the presence of an induced pulmonary edema.

With successive lung lavages, pulmonary surfactant was removed from the pig’s lungs, causing atelectasis and ARDS. Theoretically, during the course of 4 hours of ventilation, endogenous surfactant reproduction from the remaining pneumocyte type II cells could weaken the ARDS condition. Hence, the respiratory system compliance would gradually be restored. However, as demonstrated in Figure 3, the dynamic lung compliance was stable at 0.92 ml/kg/cmH_2_O for 2 hours after the therapy and even decreased to 0.6 and 0.76 ml/kg/cmH_2_O after a 10-second disconnection for the last 1.5 hours. From this, endogenous surfactant production seemed not to play a significant role in our ARDS model. However, further research on endogenous surfactant production in such animal models could be useful as background information for the ventilation management.

During the experiments, the supine position was set up and poor dorsal ventilation was expected. EIT images can be used to analyze the lung conditions; for example, improved dorsal ventilation, atelectasis or a detection of pneumothorax. This noninvasive measurement gives more insight into the lung pathophysiology during ventilation therapy at the bedside.

Considering the Berlin ARDS definition [15] and Figure 9b, after the lung lavage all of our animals were classified as severe ARDS (PaO_2_/FiO_2_ ≤ 100 mmHg). To meet the oxygenation goal, PaO_2_/FiO_2_ was improving from severe to moderate ARDS during the 4 hours of automatic ventilation therapy in most cases (PaO_2_/FiO_2_ ≤ 200 mmHg), indicating an effective performance of the automatic ventilation. Simultaneously, the plateau pressure (Pplat ≤ 30 cmH_2_O) and the pH goals were fulfilled, as shown in Figure 9c and Figure 9d, respectively.

Clinically, other adjunct therapies are available when conventional lung-protective ventilation is not sufficient: proning, nitric oxide, or extracorporeal membrane oxygenation. The most severe cases should be transferred to ARDS centers when conventional ventilation measures fail. Yet most ARDS patients have not received lung-protective ventilation [36]. The autoARDSNet protocol may thus help to foster protocol adherence.

One characteristic of this lavage-induced ARDS model, originally proposed by our group [19], is the high recruitability of the injured lung in conjunction with the concomitant changes in physiological variables (airway pressure, compliance, PaO_2_ or PaCO_2_). This model thus allowed us to maximize the changes brought about by our ventilatory protocol. Whereas the other lung injury models, such as oleic acid infusion or hydrochloric acid aspiration, may be more comparable with the human disease [37], our model is nearly ideal to study the effects of mechanical ventilation on lung injury. Hence, it would be worthwhile to also examine the performance of the closed-loop control concept in these animal models in future projects.

In clinical practice, the proposed automatic ventilation therapy system could continuously provide a standard protocol-driven ventilation for patients with ARDS. The system’s benefits include a guaranteed consistency of care, especially during night shifts or other periods of staff shortages (like epidemias). In any case, both autoARDSNet ventilation and manual intervention by clinical staff must ensure that there is no failure of measuring equipment, which is crucial in decision-making during the therapy. Reliable measurements of vital parameters are a prerequisite. At present, our system does not represent clinically applicable devices and sensors because it does not yet have any fault-tolerance measures – this must be dealt with by future manufacturers prior to any market approval by legal authorities. One should, however, emphasize that a recent study demonstrated the safety and feasibility of closed-loop ventilation in 100 patients with ARDS for 392 days [38].

## Conclusion

The primary goal of this study was to develop autoARDSNet, a prototype of fully automated ventilation therapy using the ARDSNet protocol. This protocol is known to minimize the mortality rate by 8.8% and is used in daily clinical practice worldwide. A patient with ARDS requires much attention during ventilation therapy, especially in a critical state of hypoxia. To reduce the workloads of clinical staff and to maintain the standard of ventilation therapy, automatic ventilation is a promising assistance mechanism for patients and clinical staff. Based on animal experiments, the protocol was found to be feasible and safe and can be used for patients with ARDS in the ICU.

Generally, immediate corrective action is required if the patient–ventilator interface is disconnected. In such cases, the automatic ventilation system can offer standard continuous care for the patients, thus increasing patient safety. In addition, EIT images can noninvasively be used for assessing the distribution of ventilation and monitoring other complications, such as pneumothorax, at the bedside in real time. Although the ARDSNet protocol is evidence based, it is not yet in general practice worldwide. Hence, the proposed automatic ventilation therapy system using the ARDSNet protocol may be beneficial for those centers that do not have the staff to implement the protocol, especially during night shifts, or are located in remote areas.

## Key messages

• Automatic closed-loop ventilation using the ARDSNet protocol is feasible and safe in operation.

• A patient–ventilator disconnection should be omitted by any means; but autoARDSNet ventilation was able to implement an immediate corrective and lung-protective ventilatory strategy in lavage-induced ARDS pigs.

• EIT images can be noninvasively used for assessing regional ventilation and monitoring pneumothorax at the bedside in real time.

## Abbreviations

ABG: arterial blood gas; ARDS: acute respiratory distress syndrome; ARDSNet: Acute Respiratory Distress Syndrome Network; autoARDSNet: automatic ventilation therapy using the Acute Respiratory Distress Syndrome Network; bpm: breaths per minute; EIT: electrical impedance tomography; etCO_2_: end-tidal carbon dioxide; FiO_2_: fraction of inspired oxygen; I:E ratio: inspiratory–expiratory ratio; PaCO_2_: arterial carbon dioxide tension; PaO_2_: arterial oxygen tension; PBW: predicted body weight; PEEP: positive end-expiratory pressure; P_plat_: plateau pressure; RR: respiratory rate; SaO_2_: arterial oxygen saturation; SpO_2_: peripheral oxygen saturation; V_T_: tidal volume; V_TPW_: tidal volume per weight.

## Competing interests

The authors declare that they have no competing interests.

## Authors' contributions

AP was responsible for the programming, experimental design and conceptualization and data analysis and drafted the manuscript. DS was responsible for the application for animal experiments and experimental design and conceptualization. PP and OT participated in the data analysis and interpretation. BL and SL supervised the animal experiments and involved in design and conceptualization. All authors performed the animal experiments and read, critically revised and approved the final manuscript.
